# Ultrasensitive multiplexed immunoassay of autophagic biomarkers based on Au/rGO and Au nanocages amplifying electrochemcial signal

**DOI:** 10.1038/s41598-017-02766-1

**Published:** 2017-05-26

**Authors:** Guannan Wang, Yankun Li, Jinlei Liu, Yajing Yuan, Zhaoliang Shen, Xifan Mei

**Affiliations:** 1 Department of Chemistry & The Key Laboratory for Medical Tissue Engineering of Liaoning Province, Jinzhou Medical University, Jinzhou, 121001 People’s Republic of China; 2 First Affiliated Hospital of Jinzhou Medical University, Jinzhou, 121001 People’s Republic of China; 3 The Second Hospital of Jinzhou, Jinzhou, 121001 People’s Republic of China

## Abstract

A novel sandwich-assay electrochemical immunosensor for simultaneous determination of autophagic biomarkers was introduced for the first time, the gold-reduced grapheme oxide nanocomposite (Au/r-GO) set as a good conductive platform with super high specific area, and provided more binding sites for the both antibodies of Beclin-1 and LC3B-II. While Au nanocages (AuNCs) served as good conductive platform to encapsulate a large amount of redox probe and secondary antibodies for signal amplification, due to the abundant reactive oxygen functional groups on its surface. Through differential pulse voltammetry (DPV) measurements, two separate signals can be detected directly in a single run, which represent the existence of Belin-1 and LC3B-II. Under optimized conditions, the electrochemical immunosensor exhibited good sensitivity and selectivity for the simultaneous determination of Beclin-1 and LC3B-II with linear ranges of 0.1–100 ng/mL. The detection limit for Beclin-1 and LC3B-II is 0.02 and 0.03 ng/mL respectively. This method was also applied for the analysis of Beclin-1 and LC3B-II levels in experimental cellular protein lysates, and the results were in good agreement with those of enzyme linked immunosorbent assay. This approach gives a promising simple, sensitive and quantitative strategy for the detection of autophagy

## Introduction

Recent years, lots of attentions have been focused on the “Autophagy”, which means “self-eating”in Greek, is a common degradation pathway involving the lysosomal pathway, existing in eukaryotic cells widely and as an important self-protective mechanism to maintain cell homeostasis^1–3^. At a physiological level and due to its homeostatic function, this self-eating process plays an important role in the tissue remodeling and damaged cell organelles removing. Many studies provide evidence that autophagy is crucial for several diseases e.g. Neurodegeneration, cancer initiation and progression as well as therapy resistance^3–6^.

Proteins that are usually utilized to detect the presence of autophagy. The lipidated, LC3-II, as an autophagic biomaker, is one of the few proteins that has been detected solely on the autophagosomal membrane. And Beclin-1, as another autophagic biomaker, is part of a class IIIPI3 K/Vps34 complex that induces autophagosome formation and regulates the moving of other autophagy related proteins into autophagosome. In fact, LC3-II and Beclin-1 detection are the most popular tool for examining autophagy^7^.

Commonly, the simultaneously determination of Beclin-1and LC3-II was used to indicate the occurrence of autophagy, precise and simultaneous multiplexed determination of LC3-II and Beclin-1 proteins can provide a more accurate and reliable information for detecting autophagy, with the advantages of shortened analysis time, decreased sample and simplified analytical procedure volum. Hence, it is significant to realize simultaneous determination of multianalytes in a single run. Up to now, although various of valid and effective methods have been developed for detection of autophagy related proteins LC3-II and Beclin-1^2, 8–11^, most of them require are tedious, time consuming, sophisticated operating limited sensitivity for quantitative detection and the use of labels including fluorescein and isotope.

Compared with above counterparts, in the recent year, the electrochemical immunoassays technique becomes attractive for the immunoassay of protein biomarkers, owing to its special merits, such as high sensitivity, inherent simplicity, and miniaturization^12, 13^. For development of simultaneously cover two or more biomarkers in a single run using by electrochemical immunoassay, the key difficulties are the preparation of distinguishable immunoprobes with different redox activity, especially facing the interference from blood serum substance, such as dopamine and ascorbic acid^14^. To solve this issue, some novel and high sensitive electrochemical detection strategies for proteins assay were proposed by sharing unique physicochemical properties of nanomaterials due to their extremely higher sensitivity based on stripping voltammetric detection of elemental components^15, 16^.

In order to develop enhanced immonosensors, reduced graphene oxide (rGO) is considered as an excellent support for loading electroactive species to amplify the signal due to its distinguished surface area, acceptable biocompatibility and fascinating electrocatalytic activity^17–19^. Many studies have demonstrated that rGO is a promising candidate for the electrochemical applications^20^. Meanwhile, Au nanoparticles, with their inherent biocompatibility, strong adsorption abilities, ease modification, high stability and conductivity, have also been used widely as immobilization matrices for electrochemical biosensors^21–23^. Therefore, it is expected that Au/rGO nanocomposites could exhibit enhanced sensitivities and simplify the assay system for muiti-detection of autophagy related protein LC3B -II and Beclin-1. In addition, as a kind of Au nanomaterials, Au nanocages with hollow structural and large surface area could be the idea nanocarrier for loading the redox probe to amply the electrochemical signal^24^. However, up to now, as far as we known, similar and related work have not yet been reported.

**Figure 1:**
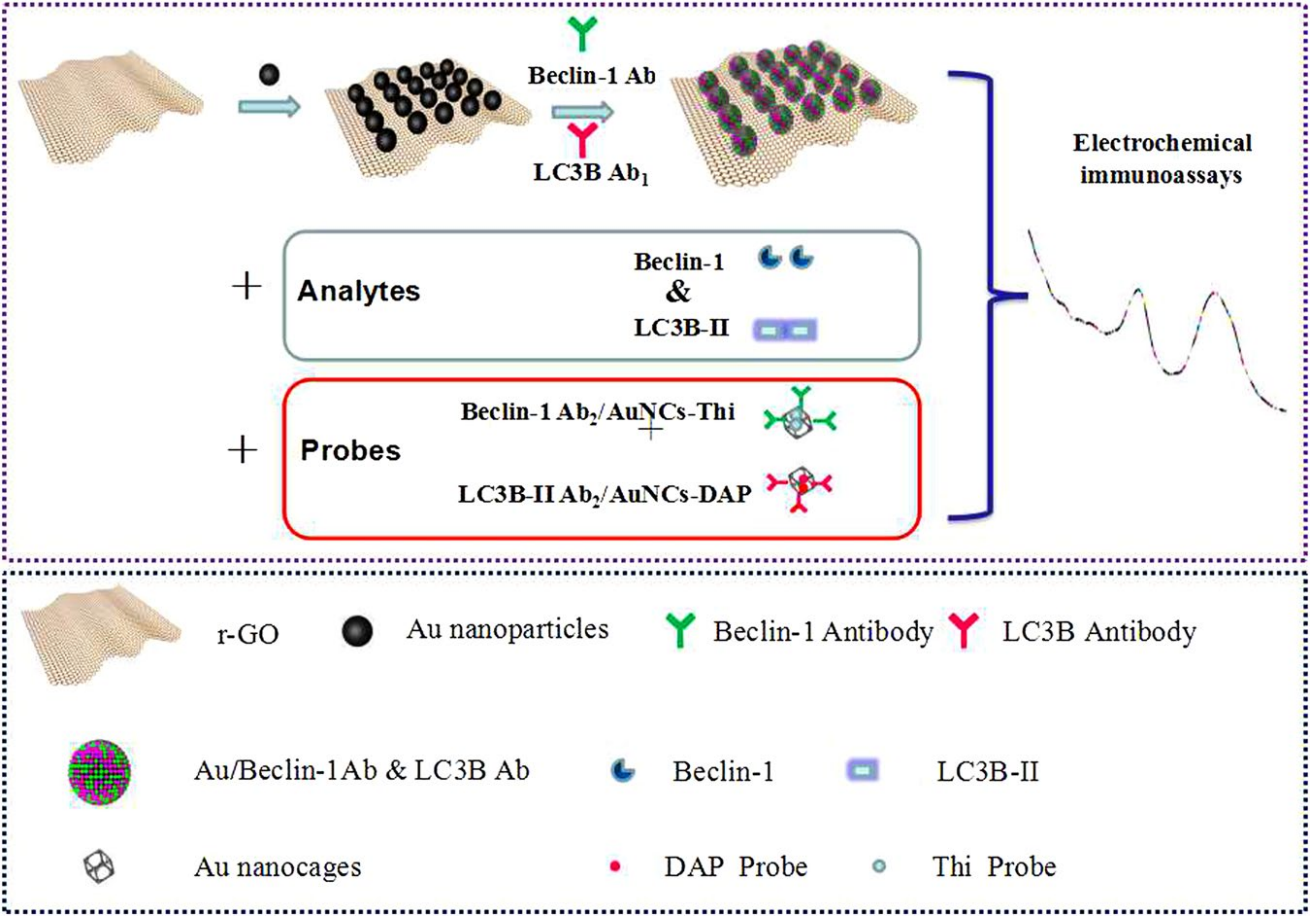
Preparation and schematic illustration of the electrochemical immunoassay protocol.

To realize the quick and ultrasensitive multiple immunoassay for simultaneous detection of LC3-II and Beclin-1 protein in a single host with lower cost and simple strategy, we developed an novel sandwich-type immunoassay biosensor based on Au/rGO platform and Au nanocages (AuNCs). Au/rGO nanocomposites set a good conductive platform with super high specific area, which also provides more fixed sites for enrichment of biomarkers proteins^25^. While AuNCs can also serve as good conductive platform to encapsulate a large amount of redox probe for signal amplification. In this study, two primary antibodies (Ab_1_) were immobilized on the surface of Au/rGO modified Au electrode (Au/rGO/Au electrode), and secondary antibodies (Ab_2_) and redox signal probe (thionin, Thi or 2,3-diaminophenazine, DPA) were conjugated with AuNCs to form immnoprobes. In the presence of target proteins (Beclin-1 and Lc3B-II), the sandwich-immuoreaction was happened among the Au/rGO/Au electrode, proteins and immnoprobes, then the immnocomplexs were formed (Fig. 1). Thereafter, the electrochemical signal was greatly increased because of the AuCNs carrying numerous redox signal molecules (Thi and DPA). The peak difference was about 0.2 mV, therefore the cointerference between LC3B-II and Beclin-1 could be neglected, indicating the immunoassay can sensitive detect two biomarkes in one run and do not interfere each other. To the best of our knowledge, the electrochemical immunoassay for simultaneous detection of autophagic two biomarkers has not been reported.

## Results and Discussion

### Characterization of the Au/rGO nanocomposites and Au NCs

As illustrated in Fig. 1, the immunoassay protocol is fabricated by the following processes: (1) The Au/rGO nanocomposites with great specific surface area and high electrical conductivity are synthesized to modified the Au electrode and capture primary Ab_1_ owing to the host-guest interaction^26^. **(**2) Au nanocages loading Thi or DAP as the redox signal tags could combine Ab_2_ directly forming Ab_2_/AuNCs-tag immunoprobe which could also be successively conjugated with the target protein via immunoreaction. (3) Once the target proteins-autophagic biomarkers Lc3B-II or/and Beclin-1 present in the test solution, then the target proteins can be captured onto the antibody sites by a specific antigen-antibody bond, which is high affinity and effective. To simultaneously detect the Lc3B-II and Beclin-1, the amplification of the electrochemical signal was employed to investigate the formation of sandwich-type immunoassays.

**Figure 2:**
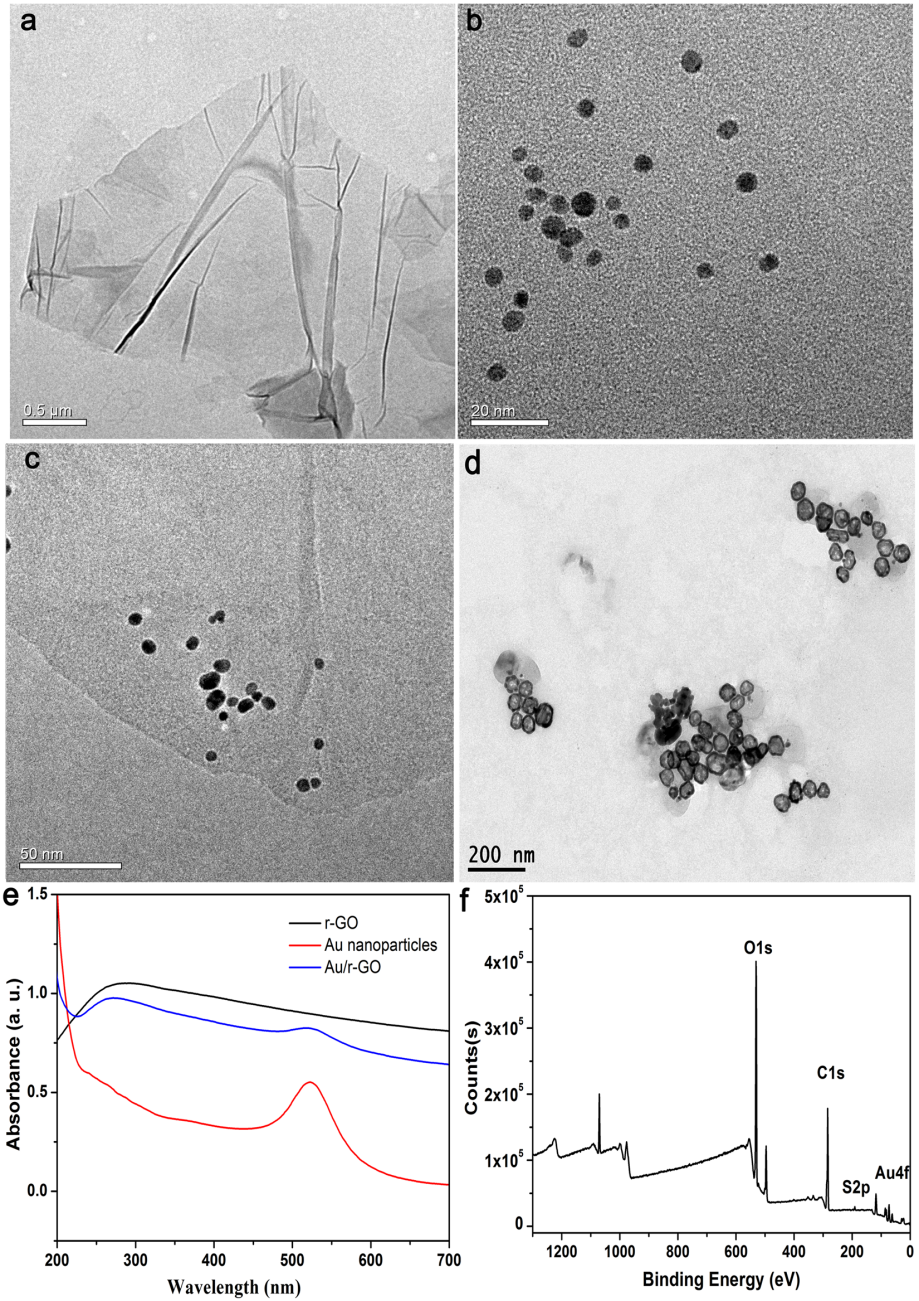
TEM images of r-GO (**a**); Au nanoparticles (**b**); Au/r-GO nanocomposites (**c**); Au nanoparticles (**d**); Ultra-vis absorption spectrum of the r-GO, Au nanoparticles and Au/r-GO nanocomposites (**e**); XPS survey spectra of the prepared Au/r-GO nanocomposites (**f**).

As described above, to clarify the formation of immunosensors and immunoprobes, several important features are discerned upon the microscopic and spectroscopic characterization. (1) A large-scale transmission electron microscopy (TEM) image (Fig. 2c) is first used to confirm the structure of Au/rGO nanocomposites, which shows Au/rGO nanocomposites are synthesized successfully with the shape of the flexible two-dimensional sheets and no evident phase separation. And compared with TEM imaging of Au nanoparticles (~5 nm) and rGO (Fig. 2a and b), we can see that a lot of Au nanoparticels with monodisperse sizes are tightly attached to the surface of rGO sheet, they are homogenously dispersed on the surface of rGO sheets without free nanoparticles formed outside the rGO sheets, which gives good evidence that Au/rGO nanocomposites is synthesized successfully, and could be a good platform for electrical biosensor. (2) The TEM imaging is also employed to characterize the morphology of the Au NCs probe. As shown in the Fig. 2d, Au NCs exhibit perfect nanocage structure with outer diameters of around 50 nm and pore size of 2–3 nm, which can provide high surface area to conjugate with Ab_2_ and load up the redox probes. (3) The UV-vis spectra were also used to confirm the formation of Au/rGO. As shown in Fig. 2e, compared with rGO, Au/rGO nanocomposites exhibit double absorption peaks at around 260 nm and 517 nm, the absorption peak at 260 nm is derived from rGO owing to the π-πtransition of aromatic C = C bonds^27^, while for the weak and broad absorption peak at 517 nm, which comes from the AuNPs. These results suggested that AuNPs were loaded onto rGO surface and the properties of rGO maintained. (4) The X-ray photoelectron spectroscopy (XPS) analysis was conducted for Au/rGO nanocomposites. As shown in Fig. 2f, C1s, O1s and Au4f can be found in the Au/rGO nanocomposites, indicating there are some oxygen groups in Au/rGO. The high resolution curve of C1s is shown in the Supplementary Fig. S1. There are three peaks at 284.6, 286.1, and 288 eV corresponding to the groups of C–C, C–O, C¼O, respectively, these reactive oxygen groups could contribute the hydroxyl group of rGO and carboxylic acid on the surface of Au nanoparticles. While the Au4f (Supplementary Fig. S2) shows the doublet peaks at 83.8 eV and 87.6 eV corresponding to the reduced Au(0) clusters and the Au(III) ions, respectively, Furthermore, there is a 1 eV red shift of XPS peak of Au(0) clusters (84.8 eV) compared with bulk Au (83.8 eV). This shift in the binding energy is typical for very small metal nanoparticles on a variety of support materials^26, 28, 29^, further confirming the presence of Au nanoparticles on the surface of rGO.

### Electrochemical properties of the immunosensor

**Figure 3:**
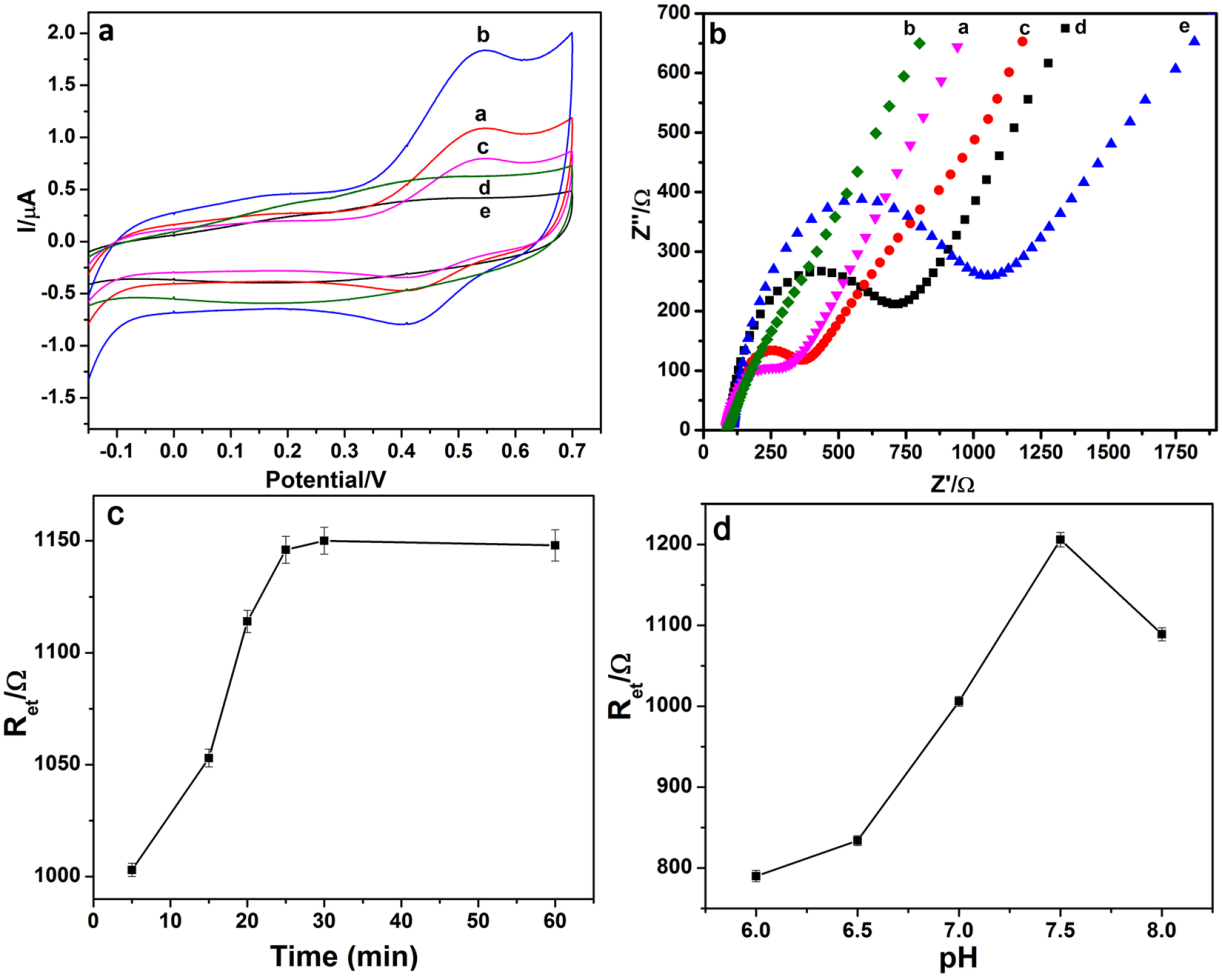
CV responses (**a**) and EIS (**b**) of the different modified electrodes in 0.1 M PBS containing 5 mM K_3_[Fe(CN)_6_]/K_4_[Fe(CN)_6_] containing 0.1 M KCl, respectively. Bare Au (curve a), electrode modified successively with Au/r-GO nanocomposites (curve b), mixture of capture anti-LC3B-II (Ab_1_) and anti-Beclin-1 (Ab_1_) (curve c),mixture of 10 ng/mL LC3B-II and Beclin-1 (curve d), mixture of anti-Beclin-1 (Ab_2_)/Au NCs and anti-LC3B-II (Ab_2_)/Au NCs (curve e). Effect of the incubation time (**c**) and pH (**d**) on the response of the immunosensor.

In this work, the rGO and Au nanoparticles with superior conductivity was used as biosensor platforms for immobilization of proteins. In order to monitor the fabrication process of the immunosensor, the cyclic voltammograms (CV) measurements of different electrode construction process were performed in 1 mM K_3_[Fe(CN)_6_] solution and normalized in Fig. 3a. Current density is obtained from the I/µA (A refers to electrode surface areas which is obtained through the Randles– Sevcik equation Ipa = 2.69 × 10^5^ *n* ^3/2^ *c*_0_ AD*R* ^1/2^ *v* ^1/2^ with CVs of different electrode modified Au electrode in 1.0 mM K_3_Fe(CN)_6_ at different scan rates from 10 to 250 mV s−1)^30^. A pair of distinct redox peaks with relatively low peak current density value from −0.2 V to 0.6 V was observed due to the oxidation and reduction of the redox couple Fe(CN)6 ^−4/−3^ on the bare Au electrode (curve a). Upon decorating with rGO/Au, the peak current density of CVs significantly increased (curve b) attributing to the increased specific surface area and excellent ability of electron transfer of the rGO/Au. When the electrode binding with the capture Ab_1_, target LC3B-II protein and LC3B-II Ab_2_/AuNCs-DAP, the peak current density of redox peaks decreased significantly step by step (curve c, d, e), this could be ascribed to form a sandwich-type immunocomplex, and the insulating layer of proteins hinders interfacial electron transfer.

On the other hand, electrochemical impedance spectrum (EIS) is an important analytical tool to understand the electron transfer kinetics in the electrode–electrolyte interface and to examine surface modifications of electrode through Bode and Nyquist complex-plane diagrams in the presence of [Fe(CN)_6_]^3−^/[Fe(CN)_6_]^4−^ as the redox probe. It also could be used to characterize the stepwise fabrication process of immunosensor. In the EIS, the diameter of the semicircle at higher frequencies corresponds to the electron-transfer resistance (Ret), which represents controls the electron-transfer kinetics of the redox probe at the electrode interface^3^. As shown in the Fig. 3b, the unmodified bare Au electrode depicted a smaller semicircle implies a nice smaller resistance. After rGO/Au nanoparticles modified, the electrode has a significantly promote electron transfer, and obviously straight line was obtained with ignorable semicircle due to Au nanoparticles suggesting that synergistic effects of Au and rGO in the nanocomposites are promising for providing better electron transfer pathway. With the immobilization with capture Ab_1_, target LC3B-II protein and LC3B-II Ab_2_/AuNCs-DAP, the Ret increased step by step, indicating a high electron transfer resistance, because the movement of the [Fe(CN)_6_]^3-/4-^ towards electrode surface are repulsion and prevented by the immunocomplexes layers, which is in good agreement with the results obtained from CVs. Based on above results, the sandwich-type immunoreaction was proved and simultaneous detection of autophagic biomarkers LC3B-II and Beclin-1 was possible.

### Optimization of analytical condition

To verify the optimal conditions, we optimized two experimental details. The incubation time was a important influence factor because the incubation time of antibody with target protein had great effect on sandwich-type immunoreaction and fabrication of immunocomplexes. The effects of incubation time on the EIS responses were shown in Fig. 3c, the results showed that the R_et_ response gradually increased as the incubation time from 5 to 30 min, and then unchanged almost as the incubation time increased above 30 min. Therefore, 30 min was selected as the optimal incubation time for the detection. In addition, the pH of detection solution is also one of the most important factors. Unsuitable pH not only change antibodies activity, but also affects the immunosensor electrochemical performance^14^. As shown in Fig. 3d, the R_et_ response increased with pH value from 6.0 to 7.5, however, it decreased at higher pH condition, which indicate the high pH condition affects the activity of antibody and cause the binding between the antibody and immunosensor weakened. In order to get a well sensitivity for following measurements, PBS of pH 7.5 was chosen as the detection solution for simultaneous determination of Belin-1 and LC3B-II.

### Evaluation of cross-reactivity, specificity, reproducibility and stability

**Figure 4:**
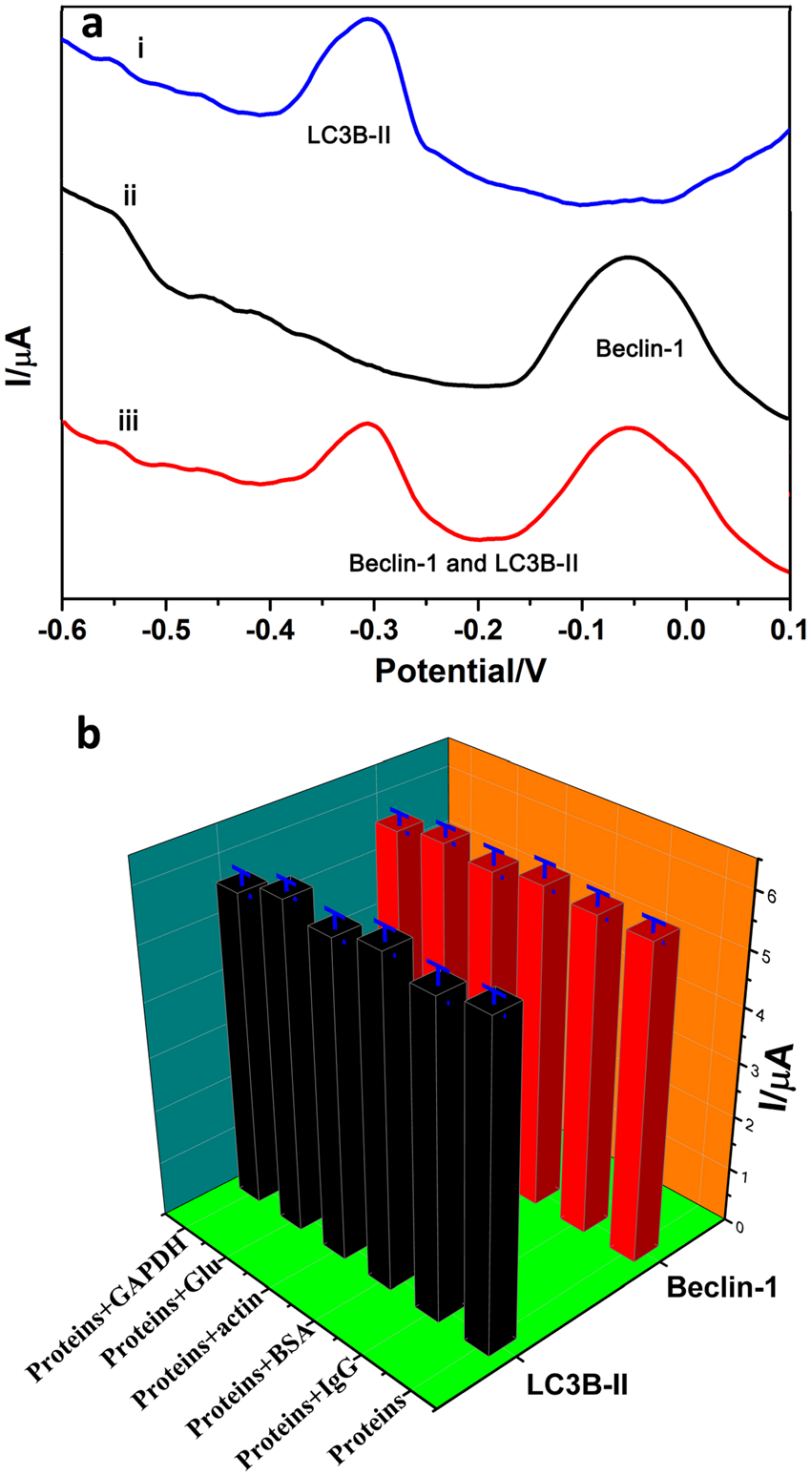
DPV responses of immunosensor in presence of various conditions (**a**): 10 ng/mL LC3B-II and 0 ng/mL Beclin-1(i); 0 ng/mL LC3B-II and 10 ng/mL Beclin-1(ii); 10 ng/mL LC3B-II and 10 ng/mL Beclin-1(iii).Specificity of the immunosensor (**b**): current response of the immunosensor to 10 ng/mL LC3B-II and Beclin-1, and 100 ng/mL interfering substances of IgG, BSA, Actin, Glucose and GAPDH.

Three control tests were carried out to evaluate the cross-reactivity of the multiplexed electrochemical immunosensor under the same experimental conditions. The tests were as follows: (1) 10.0 ng/mL Belin-1 was assayed; (2)10.0 ng/mL LC3B-II was assayed; (3) 10.0 ng/mL Belin-1 and 10.0 ng/mL LC3B-II were simultaneously monitored using the proposed method. The results were shown in Fig. 4a. From curve i and ii, the corresponding target analyte showed a higher current response, meanwhile, another one exhibited an extremely low current shift, which indicated that the interference with each other was low, and the cross-reactivity was negligible, When 10.0 ng/mL Belin-1 and 10.0 ng/mL LC3B-II were simultaneously monitored, the stripping peak of Thi and DAP were simultaneously observed (curve iii) with similar results with curve a and b in stripping peak position and current response.

In order to simulate the interferences from cellular protein lysates and evaluate the specificity of the immunosensor, the specificity of the immuosensor was also tested, and IgG, BSA, actin, glucose and GAPDH (glyceraldehyde-3-phosphate dehydrogenase) were chosen as possible interfering substances. 10 ng/mL Belin-1 and 10.0 ng/mL LC3B-II were first mixed with 100 ng/mL of IgG, BSA, actin, glucose and GAPDH respectively. Then the current response of immunosensor was recorded and shown in Fig. 4b. Compared with the control (pure 10 ng/m Belin-1 and 10.0 ng/mL LC3B-II), the variation in current of immunosensor caused by the interference substances was weak without significant differences (p > 0.05), indicating that the immunosensor possesses good specificity for Belin-1 and LC3B-II.

The reproducibility of the immunosensor was also investigated via a standard sample containing 5 ng/mL Belin-1 and LC3B-II solutions was measured five times using five freshly prepared immunosensor. The relative standard deviation (RSD) of the five measurements was calculated to be was 9.1%, 8.2%, 6.7% 7.2% and 8.6%, respectively, suggesting the proposed immunosensor possessed acceptable precision and reproducibility. In addition, the stability of immunosensor was also evaluated. After stored in refrigerator at 4 °C for two weeks, the 5 ng/mL Belin-1 and LC3B-II solutions was measured, over 91.5% of the electric signal remained compared with the freshly prepared at the end of fourteenth day, indicating the stability of proposed immunosensor was acceptable.

In brief, we determined that the proposed method exhibited low interference between the two analytes, good specificity, reproducibility and stability.

### Simultaneous electrochemical determination of Belin-1 and LC3B-II

**Figure 5:**
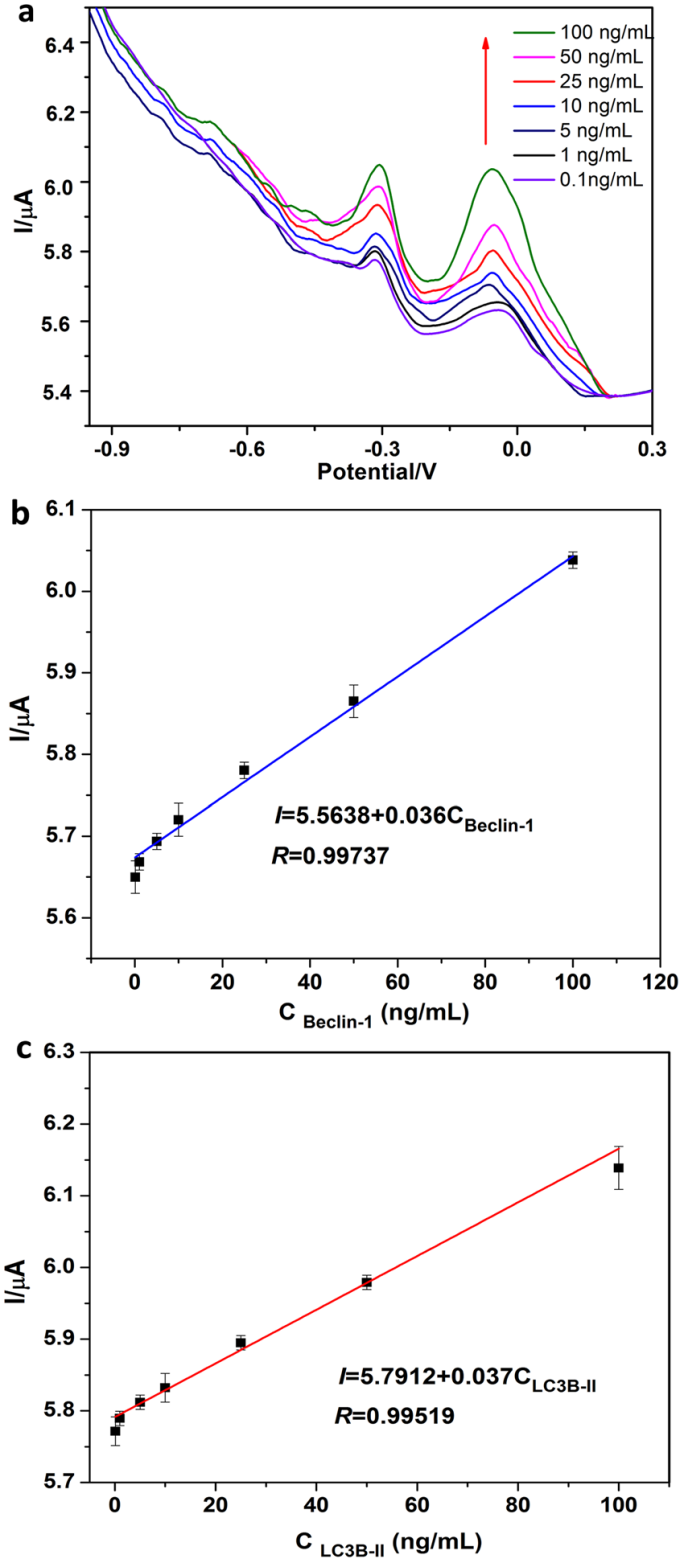
DPV responses of the proposed immunosensor after incubation with different concentrations of LC3B-II and Beclin-1 (**a**); Calibration curves of the multiplexed immunoassay toward Beclin-1 (**b**) and LC3B-II (**c**) in PBS (pH 7.5).

Under optimized assay conditions, the performance of the proposed immunosensor was evaluated using different concentrations of Belin-1 and LC3B-II mixture. As shown in Fig. 5, the reduction peaks of Thi and DPA at near −0.05 V and −0.35 V represent the existence of Belin-1 and LC3B-II, and were proportional to the concentration of Belin-1 and LC3B-II in the incubation solution. The DPV peak currents of the Thi and DPA for simultaneous detection of Belin-1 and LC3B-II increased with the increment of concentrations of Belin-1 and LC3B-II in the incubation solution. In the ranges of 0.1–100 ng/mL for both of Belin-1 and LC3B-II, the calibration plots displayed a good linear relationship between the voltammetric peaks currents and the concentration of analytes. The correlation coefficient of Belin-1 and LC3B-II was 0.997 and 0.995 separately, and the detection limit was for Belin-1 and LC3B-II is 0.02 and 0.03 ng/mL. The sensitively and linear range was acceptable compared with the enzymatic reaction enhanced immunoassay (0.01 ng/mL)^14^. It is clear that simultaneous determination of autophagic proteins Belin-1 and LC3B-II was achieved on proposed immnosensor with high sensitivity and selectivity. The results indicated that the proposed electrochemical immnoassay exhibited a satisfactory electrochemical performance and possessed an acceptable wide linear range and low limit.

### Application

In order to evaluate the application of the proposed electrochemical immunoassay, three spiked samples of experimental cellular protein lysates with different concentrations of Belin-1 and LC3B-II were tested by using proposed immunoassay and ELISA for reference, respectively. The analytical results were shown in Table 1. The spiked recovery was in the range of 92–102% by our proposed electrochemical immunosensor. These results indicated that proposed electrochemical immunosensor was feasibility and accuracy enough in scientific research for simultaneous determination of autophagic proteins Belin-1 and LC3B-II.

**Table 1:** Comparison of the assay results for spiked samples by using the proposed immunosensor and the referenced ELISA method. (n = 5).

Sample	Added (ng/mL)		Immunosensor (ng/mL)		ELISA (ng/mL)		Recovery (%)	
	Beclin-1	LC3B-II	Beclin-1	LC3B-II	Beclin-1	LC3B-II	Beclin-1	LC3B-II
1	0.5	0.5	0.46 ± 0.03	0.48 ± 0.05	—	—	92%	96%
2	5.0	5.0	4.7 ± 0.1	5.1 ± 0.05	4.9 ± 0.8	4.6 ± 0.9	94%	102%
3	10.0	10.0	10.0 ± 0.5	9.6 ± 0.4	10.2 ± 1.2	10.1 ± 0.8	100%	96%

^a^The symbol“–”suggest that the sample could not be detected by the corresponding method.

## Conclusions

In summary, we have developed a simple and reliable electrochemical immunosensor for simultaneous detection of autophagic proteins Belin-1 and LC3B-II based on r-GO/Au nanocomposites modification Au electrode and Au nanocages loaded with Thi and DAP as signal enhancers. The distinct voltammetric peaks of Thi and DAP which had a close relationship with each corresponding biomaker can be detected through DPV in a single run. Moreover, Au, r-GO and Au nanocages with its exceptionally high surface area, good conductivity and biocompatibility was used as an excellent carrier for immobilizing antibodies and further loading a large amount of redox probes, which greatly increase the probability of antibody-antigen interactions and beneficial for improving sensitivity of the immunosensor. The proposed immunosensor showed excellent analytical performance for developing simultaneous multiplexed analyses of a panel of targets.

## Materials and Methods

### Reagents and apparatus

Reduced graphene oxide (rGO) sheet was purchased from XF Nanomaterials Technology Co., Ltd.(Nanjing, China). Anti-Beclin-1 (Ab_1_) antibody, Anti-Beclin-1 (Ab_2_) antibody, anti-LC3B-II antibody (Ab_1_), Anti-LC3B-II antibody Mouse monoclonal (Ab_2_), Beclin-1 protein and LC3B-II protein were purchased from Sigma-Aldrich Co., Ltd. (United States). Hydrogen tetrachloroaurate (III) bydrate (HAuCl_4_), KCl, albumin from bovine serum (BSA), NH_3_·H_2_O, N,N-dimethylformamide (DMF), potassium ferricyanide (K_3_Fe(CN)_6_) and potassium ferrocyanide (K_4_Fe(CN)_6_) were provided by Sigma-Aldrich Co., Ltd. (United States). Phosphate buffered saline (PBS, 0.01 M) at different pH was prepared by mixing stock solutions of NaH_2_PO_4_ and Na_2_HPO_4_, and the pH was adjusted by NaOH and H_3_PO_4_. Twice-quartz-distilled water was used in this study.

All electrochemical experiments of cyclic voltammetry (CV), differential pulse voltammetry (DPV) and electrochemical impedance spectroscopy (EIS) were performed on a CHI650D electrochemical workstation (Shanghai Chenhua Equipment, China) with a conventional three-electrode system using a modified gold electrode as the working electrode, a platinum wire electrode as the counter electrode, and a saturated Ag/AgCl (KCl) as the reference electrode. Transmission electron microscope (TEM) images were recorded using a FEI TECNAI G20 high-resolution transmission electron microscope operating at 200 kV. The samples were prepared by depositing a drop of a diluted colloidal solution on a carbon grid and allowing the liquid to dry in air at room temperature. Raman spectra were performed by Laser confocal micro-raman spectroscopy (LabRAM HR800). UV–vis measurements were carried out at room temperature on a UV-2550UV–vis spectrophotometer (Shimadzu,Japan). And The binding energy scales of the spectra for XPS measurements using a model Thermo ESCALAB 250XI.

### Preparation of Au/rGO nanocomposites and Au nanocages

The Au/rGO nanocomposites were fabricated using the one-step method according to the Tang’s method with slight revision^25^. In a typical, 100 μL of 50 mM HAuCl_4_ solution was add to 10 mL of 0.17 mg/mL rGO sheets solution which had been mixed in 5 mL NH_3_·H_2_O and 5 mL DMF at room temperature with an initial ultrasonic procedure. After continuous ultrasonic for 10 min, the resulting products were collected by centrifugation and washed several times with pure water to remove residual NH_3_·H_2_O and DMF. Finally the Au/rGO nanocomposites products were obtained by a freeze drying method and stored in darkness at 4 °C. For the next experiment, DMF was used to disperse Au/rGO nanocomposites due to its proper polarity and relatively lower boiling point. 2 mg Au/rGO nanocomposites was dispersed in 1 mL of DMF by 1 h of ultrasonication to form an Au/rGO stock solution.

Mercaptosuccinic acid functionalized Au nanocages (AuNCs) of mean 50 nm size together with a pore size of 2–3 nm were prepared according to previous method using Ag nanocubes as sacrificial templates with minor revesion^24^. And last, the carboxylic acid functionalized Au nanocages were synthesized and dispersed in ultrapure water (1 mg/mL) for further use

### Preparation of distinguishable signal immunoprobes

Anti-Beclin-1 (Ab_2_) and Anti-LC3B-II (Ab_2_) were conjugated to the carboxylic acid functionalized Au nanocages via NHS/EDC process, separately. First, 1 mL of as-prepared AuNCs was activated by 0.1 mol/L freshly prepared NHS and EDC solution at room temperature with stirring for one hour. Then, 100 μL of Ab_2_ (10 μg/mL) solution was added to the solution and mixed well on a rotary shaker overnight at room temperature. After the conjugation and centrifugation, the Triton-X 100 (0.25% (w/v), 5 mL) and BSA (1% (w/v), 5 mL) were added with rotary shaking for another one hour. Finally, the resulting Ab_2_ functionalized AuNCs (Ab_2_/AuNCs) were separated from free biomolecules by gel filtration using Sephacryl HR-300 gel medium. Then the Beclin-1 Ab_2_/AuNCs and LC3B-II Ab_2_/AuNCs were prepared.

To load the redox probes, 1 mL of Ab_2_/AuNCs were mixed with 100 μL of 20 mM Thi or DAP, respectively. In this work, the Beclin-1 Ab_2_/AuNCs were used to loaded up Thi, and the LC3B-II Ab_2_/AuNCs was loaded up DAP. After 5 h stirring under room temperature and subsequently centrifugation and rosined by ultrapure water for several times, both conjugated probes Beclin-1 Ab_2_/AuNCs-Thi, and LC3B-II Ab_2_/AuNCs-DAP were obtained owing to the strong electrostatic binding, and re-dispersed in 4 mL ultrapure water.

### Fabrication of the immunosensor

Prior to modification, three gold electrodes (2 mm in diameter) were sequentially polished repeatedly with 0.3 μm and 0.05 μm alumina powders and rinsed thoroughly with pure water after each step. Then, the electrodes were sonicated in ethanol and ultrapure water, respectively, to remove the excess alumina powders and organic contaminants on the electrode surface. After drying with nitrogen, 20 μL of the Au/rGO nanocomposites dispersion was dropped by a pipette onto the surface of the pretreated gold electrodes and allowed to dry in a vacuum desiccators for 1 h, then a thin membrane was formed on the surface of electrodes to get the Au/rGO/Au electrodes. Next, 20 μL of 10 μg/mL primary antibody (Ab_1_) mixture solution (1:1 Beclin-1 Ab_1_ and LC3B-II Ab_1_) was dropped on the surface of Au/rGO/Au electrodes and kept for 12 h in a refrigerator (Notes:Ab_1/_Au/rGO/Au electrodes). After that, it was incubated with 1 wt% BSA solution (in 0.01 M PBS, pH7.5) to prevent nonspecific adsorption between the antigen and the electrode surface, followed by rinsing with washing buffer for further use.

Similarly, Beclin-1 Ab_1/_Au/rGO/Au electrodes and LC3B-II Ab_1/_Au/rGO/Au electrodes bioconjugates were synthesized according to the similar procedures above.

### Sandwich-type electrochemical determination of Beclin-1 and LC3B-II

For the sandwich-type electrochemical determination, the Ab_1/_Au/rGO/Au electrodes were incubated with 500 μL of various concentrations of corresponding protein solution of Beclin-1, LC3B-II and mixed proteins for 1 h at room temperature, respectively. Then the electrodes were washed extensively to remove unbounded protein. Subsequently, it was further incubated as-prepared immunoprobe (Beclin-1 Ab_2_/AuNCs-Thi and LC3B-II Ab_2_/AuNCs-DAP) for another 30 min at 37 °C. Then the protein was well immobilized with its antibody (Ab_1_ and Ab_2_) on the modified gold electrode surface by the specific immunobinding. After rinsed thoroughly with PBS buffer solution, DPV measurements were performed in the PBS buffer solution between −0.6 to 0.1 V with pulse amplitude of 50 mV.

### Statistical analysis

The whole data were appeared in this study as mean ± standard deviation, and the significance was affirmed using one-way analysis of variance. Difference were taken into account statistically significant at p < 0.05. Statistical analysis was computed using SPSS 22.0 software. The origin 9.0 software was used to do statistical analysis of Figs 4b, 5b,c, and the limit of detection (LOD) for Beclin-1 and LC3B-II was calculated using the 3*sigma method (three times standard deviation of the linear plot)^31^.

## Electronic supplementary material

Ultrasensitive multiplexed immunoassay of autophagic biomarkers based on Au/rGO and Au nanocages amplifying electrochemcial signal
