# Supplementary material for: Ultrasensitive multiplexed immunoassay of autophagic biomarkers based on Au/rGO and Au nanocages amplifying electrochemcial signal
Source: Sci Rep. 2017 May 26;7:2442. doi: 10.1038/s41598-017-02766-1 (PMC5446417; doi:10.1038/s41598-017-02766-1)
Supplement: Supplementary file 1 — Ultrasensitive multiplexed immunoassay of autophagic biomarkers based on Au/rGO and Au nanocages amplifying electrochemcial signal [file 41598_2017_2766_MOESM1_ESM.doc]

Ultrasensitive multiplexed immunoassay for autophagic biomarkers based on Au/rGO and Au nanocages amplifying electrochemcial signal

Guannan Wang,^a*^ Yankun Li,^a^ Jinlei Liu,^b^ Yajing Yuan^b^ Zhaoliang Shen^c^ & Xifan Mei ^a,b*^

*^a^Department of Chemistry&The Key Laboratory for Medical Tissue Engineering of Liaoning Province, Jinzhou Medical University, Jinzhou, 121001,People’s Republic of China.*

*^b^First Affiliated Hospital of Jinzhou Medical University, Jinzhou, 121001, People’s Republic of China.*

*^c^The Second Hospital of Jinzhou, Jinzhou, 121001, People’s Republic of China.*

*Correspondence authors should be addressed to G. W.(email:chemwangguannan@gmail.com); X. M. (email: meixifan1971@163.com)*


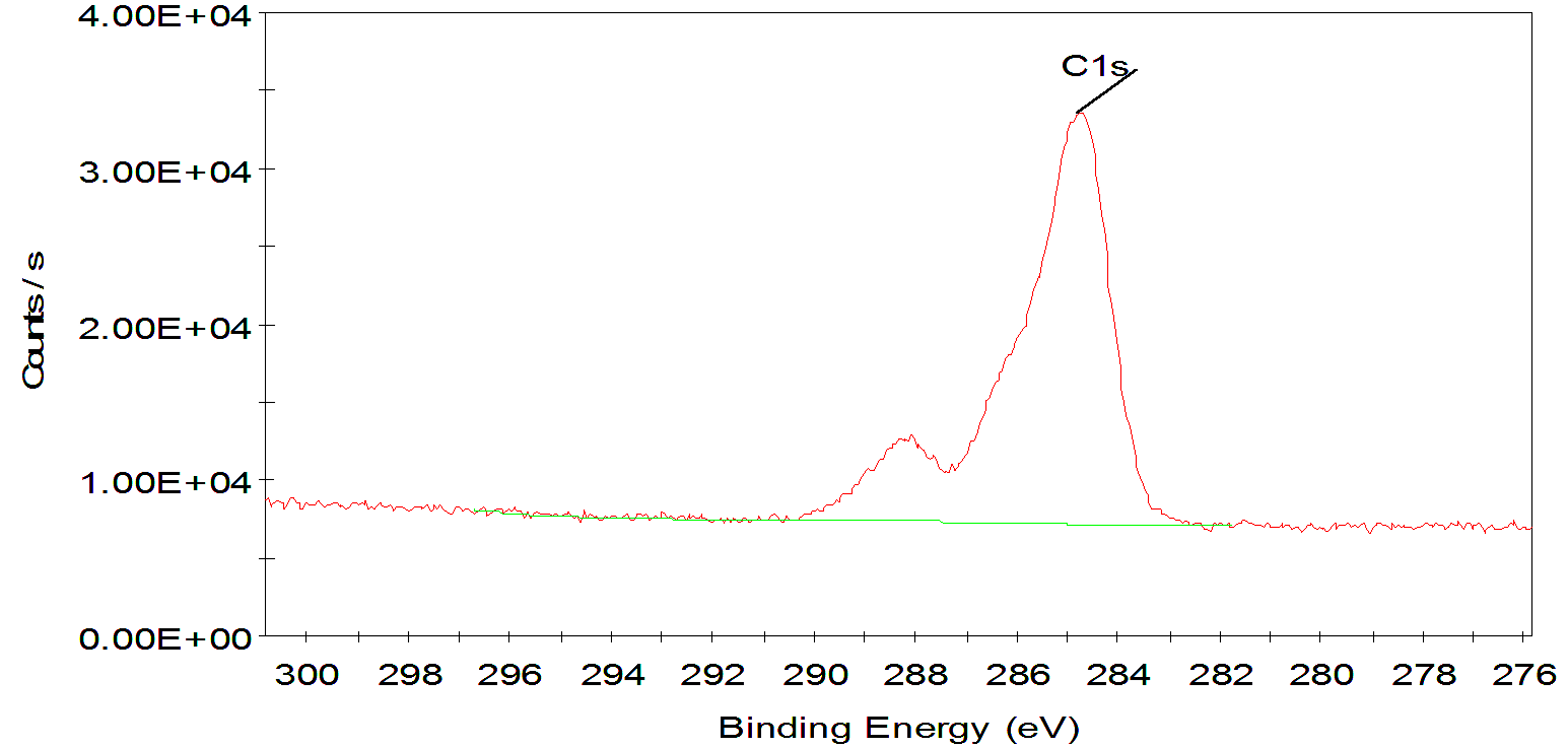


**Fig.S1** High resolution XPS of C1s peak


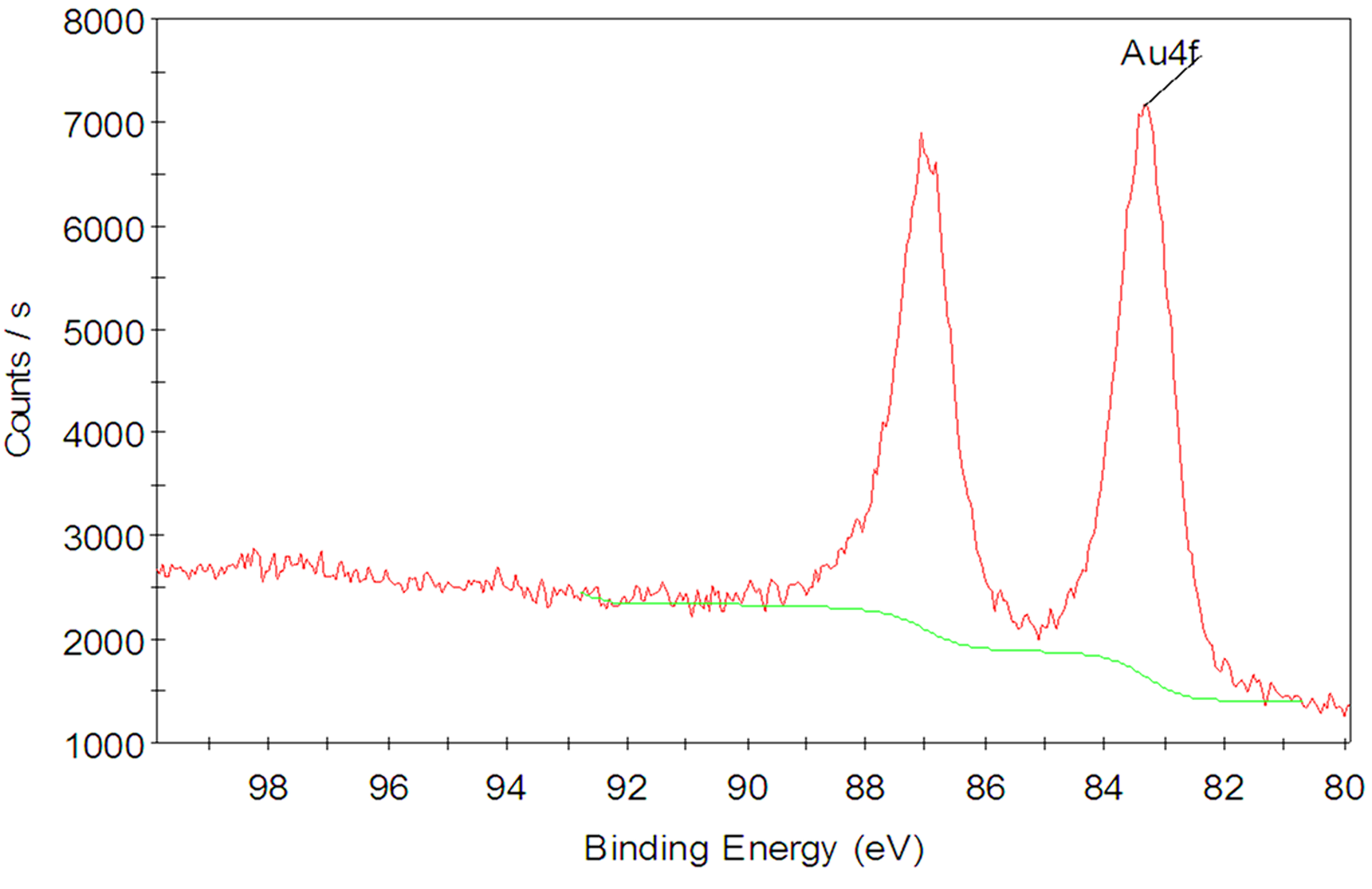


**Fig.S2**  High resolution XPS of Au4f peak
